# Cancer of the ovary, fallopian tube, and peritoneum: 2021 update

**DOI:** 10.1002/ijgo.13878

**Published:** 2021-10-20

**Authors:** Jonathan S. Berek, Malte Renz, Sean Kehoe, Lalit Kumar, Michael Friedlander

**Affiliations:** ^1^ Stanford Women’s Cancer Center Stanford Cancer Institute Stanford University School of Medicine Stanford California USA; ^2^ Oxford Gynecological Cancer Center Churchill Hospital Oxford UK; ^3^ St Peter’s College Oxford UK; ^4^ Department of Medical Oncology All India Institute of Medical Sciences New Delhi India; ^5^ Royal Hospital for Women Sydney Australia; ^6^ Prince of Wales Clinical School University of New South Wales Sydney Australia

**Keywords:** cancer staging, chemotherapy, fallopian tube, FIGO Cancer Report, ovarian, ovary, peritoneum

## Abstract

In 2014, FIGO’s Committee for Gynecologic Oncology revised the staging of ovarian cancer, incorporating ovarian, fallopian tube, and peritoneal cancer into the same system. Most of these malignancies are high‐grade serous carcinomas (HGSC). Stage IC is now divided into three categories: IC1 (surgical spill); IC2 (capsule ruptured before surgery or tumor on ovarian or fallopian tube surface); and IC3 (malignant cells in the ascites or peritoneal washings). The updated staging includes a revision of Stage IIIC based on spread to the retroperitoneal lymph nodes alone without intraperitoneal dissemination. This category is now subdivided into IIIA1(i) (metastasis ≤10 mm in greatest dimension), and IIIA1(ii) (metastasis >10 mm in greatest dimension). Stage IIIA2 is now “microscopic extrapelvic peritoneal involvement with or without positive retroperitoneal lymph node” metastasis. This review summarizes the genetics, surgical management, chemotherapy, and targeted therapies for epithelial cancers, and the treatment of ovarian germ cell and stromal malignancies.

## Synopsis

The 2014 FIGO staging incorporates ovarian, fallopian tube, and peritoneal cancer into the same system. This review summarizes the updated staging and treatment of these malignancies.

## 1 INTRODUCTION

### 1.1 Primary sites: ovarian, fallopian tube, and peritoneal cancer

In 2014, FIGO’s Committee for Gynecologic Oncology revised the staging to incorporate ovarian, fallopian tube, and peritoneal cancer in the same system. Changing the staging system required extensive international consultation. The primary site (i.e. ovary, fallopian tube, or peritoneum) is designated, where possible. When it is not possible to clearly delineate the primary site, these should be listed as “undesignated”.^1^, ^2^

It has been presumed that fallopian tube malignancies were rare.^2^ However, histologic, molecular, and genetic evidence shows that as many as 80% of tumors that were classified as high‐grade serous carcinomas of the ovary or peritoneum may have originated in the fimbrial end of the fallopian tube.^3^, ^4^, ^5^, ^6^, ^7^, ^8^ Therefore, the incidence of fallopian tube cancers may have been substantially underestimated. These new data support the view that high‐grade serous ovarian, fallopian tube, and peritoneal cancers should be considered collectively, and that the convention of designating malignancies as having an ovarian origin should no longer be used, unless that is clearly the origination site. It has been suggested that extrauterine tumors of serous histology arising in the ovary, fallopian tube, or peritoneum might be described collectively as “Müllerian carcinomas”^1^, ^2^ or “pelvic serous carcinomas”.^9^ The latter tumor designation is controversial because some peritoneal tumors might arise in extrapelvic peritoneum. Therefore, the simple term “serous carcinoma" is preferred, and most of these are high‐grade serous carcinomas (HGSC).

Although there has been no formal staging for peritoneal cancers, the FIGO staging system is used with the understanding that it is not possible to have a Stage I peritoneal cancer.

#### 1.1.1 Primary site

Ovarian epithelial tumors may arise within endometriosis or cortical inclusions of Müllerian epithelium, likely a form of endosalpingiosis. These include low‐grade endometrioid carcinomas, clear cell carcinomas, borderline and low‐grade serous carcinomas, and mucinous carcinomas. These tumors are thought to evolve slowly from lower‐grade precursor conditions (endometriotic cysts, cystadenomas, etc) and are classified as type I tumors.^5^ Fallopian tube carcinomas arise in the distal fallopian tube and the majority of these are high‐grade serous carcinomas. These are thought to evolve rapidly from more obscure precursors and are designated as type II tumors.^5^, ^6^ This latter group encompasses high‐grade endometrioid carcinomas and carcinosarcomas. All of these high‐grade carcinomas are nearly always associated with mutations in the *TP53* gene.^5^

#### 1.1.2 Lymphatic and lymph node drainage

The lymphatic drainage of the ovaries and fallopian tubes is via the utero‐ovarian, infundibulopelvic, and round ligament pathways and an external iliac accessory route into the following regional lymph nodes: external iliac, common iliac, hypogastric, lateral sacral, para‐aortic lymph nodes and, occasionally, to the inguinal nodes.^1^, ^10^, ^11^, ^12^ The peritoneal surfaces can drain through the diaphragmatic lymphatics and hence to the major venous vessels above the diaphragm.

#### 1.1.3 Other metastatic sites

The peritoneum, including the omentum and pelvic and abdominal viscera, is the most common site for dissemination of ovarian and fallopian tube cancers. This includes the diaphragmatic and liver surfaces. Pleural involvement is also seen. Other extraperitoneal or extrapleural sites are relatively uncommon, but can occur.^1^, ^10^, ^11^, ^12^ After systematic pathologic analysis has excluded a tubal or ovarian site of origin, malignancies that appear to arise primarily on the peritoneum have an identical spread pattern, and frequently may involve the ovaries and fallopian tubes secondarily. These “peritoneal” tumors are thought to arise in endosalpingiosis.

### 1.2 Classification rules

Although CT scans can delineate the intra‐abdominal spread of disease to a certain extent, ovarian, fallopian tube, and peritoneal cancers should be staged surgically. Operative findings determine the precise histologic diagnosis, stage, and therefore the prognosis, of the patient.^1^, ^9^, ^10^, ^12^, ^13^, ^14^

In selected patients with advanced stage disease, it may be appropriate to initiate chemotherapy prior to surgical intervention, and in these cases there should be histologic or cytologic confirmation of the diagnosis prior to starting neoadjuvant chemotherapy (see 5.2.2. below).

Chest radiograms may serve as a screen for pleural effusions. As distant metastases are infrequent, there is no requirement for other radiological evaluation unless symptomatic. Serum CA125 levels may be useful in determining response to chemotherapy, but they do not contribute to staging.

#### 1.2.1 Fallopian tube involvement

Fallopian tube involvement can be divided into three categories. In the first, an obvious intraluminal and grossly apparent fallopian tube mass is seen with tubal intraepithelial carcinoma (carcinoma in situ) that is presumed to have arisen in the fallopian tube. These cases should be staged surgically with a histologic confirmation of disease. Tumor extension into the submucosa or muscularis and to and beyond the serosa can therefore be defined. These features, together with the laterality and the presence or absence of ascites, should all be taken into consideration.^1^, ^3^, ^6^, ^7^

In the second scenario, a widespread serous carcinoma is associated with a tubal intraepithelial carcinoma. A visible mass in the endosalpinx may not be seen but the histologic findings should be noted in the pathology report since they may indicate a fallopian tube primary. Tumors obliterating both fallopian tube and ovary may belong to this group but whether a presumptive assignment of a tubal origin can be made in such cases is controversial given that tubal intraepithelial carcinoma cannot be confirmed.

In the third scenario—risk‐reducing salpingo‐oophorectomy—tubal intraepithelial carcinoma may be the only finding. It should be reported as originating in the fallopian tube and managed accordingly. The majority of early serous cancers detected are found in the fallopian tube, irrespective of genetic risk.^15^, ^16^

#### 1.2.2 FIGO staging

The updated, revised FIGO staging system combines the classification for ovarian, fallopian tube, and peritoneum cancer. It is based on findings made mainly through surgical exploration (as outlined above). Table 1 presents the 2014 FIGO staging classification for cancer of the ovary, fallopian tube, and peritoneum. The equivalents within the Union for International Cancer Control (UICC) TNM classification are presented in Table 2.

**TABLE 1:** FIGO staging classification for cancer of the ovary, fallopian tube, and peritoneum

Stage I: Tumor confined to ovaries or fallopian tube(s)	T1‐N0‐M0
IA: Tumor limited to 1 ovary (capsule intact) or fallopian tube; no tumor on ovarian or fallopian tube surface; no malignant cells in the ascites or peritoneal washings	**T1a‐N0‐M0**
IB: Tumor limited to both ovaries (capsules intact) or fallopian tubes; no tumor on ovarian or fallopian tube surface; no malignant cells in the ascites or peritoneal washings	**T1b‐N0‐M0**
IC: Tumor limited to 1 or both ovaries or fallopian tubes, with any of the following:	
IC1: Surgical spill	**T1c1‐N0‐M0**
IC2: Capsule ruptured before surgery or tumor on ovarian or fallopian tube surface	**T1c2‐N0‐M0**
IC3: Malignant cells in the ascites or peritoneal washings	**T1c3‐N0‐M0**
**Stage II: Tumor involves 1 or both ovaries or fallopian tubes with pelvic extension (below pelvic brim) or peritoneal cancer**	**T2‐N0‐M0**
IIA: Extension and/or implants on uterus and/or fallopian tubes and/or ovaries	**T2a‐N0‐M0**
IIB: Extension to other pelvic intraperitoneal tissues	**T2b‐N0‐M0**
**Stage III: Tumor involves 1 or both ovaries or fallopian tubes, or peritoneal cancer, with cytologically or histologically confirmed spread to the peritoneum outside the pelvis and/or metastasis to the retroperitoneal lymph nodes**	**T1‐3/N0‐1/M0**
IIIA1: Positive retroperitoneal lymph nodes only (cytologically or histologically proven):	**T1/T2‐N1‐M0**
IIIA1(i) Metastasis up to 10 mm in greatest dimension	
IIIA1(ii) Metastasis more than 10 mm in greatest dimension	
IIIA2: Microscopic extrapelvic (above the pelvic brim) peritoneal involvement with or without positive retroperitoneal lymph nodes	**T3a2‐N0/N1‐M0**
IIIB: Macroscopic peritoneal metastasis beyond the pelvis up to 2 cm in greatest dimension, with or without metastasis to the retroperitoneal lymph nodes	**T3b‐N0/N1‐M0**
IIIC: Macroscopic peritoneal metastasis beyond the pelvis more than 2 cm in greatest dimension, with or without metastasis to the retroperitoneal lymph nodes (includes extension of tumor to capsule of liver and spleen without parenchymal involvement of either organ)	**T3c‐N0/N1‐M0**
**Stage IV: Distant metastasis excluding peritoneal metastases**	**Any T, any N, M1**
Stage IVA: Pleural effusion with positive cytology	
Stage IVB: Parenchymal metastases and metastases to extra‐abdominal organs (including inguinal lymph nodes and lymph nodes outside of the abdominal cavity)	

**TABLE 2:** Cancer of the ovary, fallopian tube and peritoneum: FIGO staging (2014) compared with TNM classification^a^

FIGO (Designate primary: Tov, Tft, Tp, or Tx)	UICC		
	T	N	M
Stage			
IA	T1a	N0	M0
IB	T1b	N0	M0
IC	T1c	N0	M0
IIA	T2a	N0	M0
IIB	T2b	N0	M0
IIIA	T3a	N0	M0
	T3a	N1	M0
IIIB	T3b	N0	M0
	T3b	N1	M0
IIIC	T3c	N0−1	M0
	T3c	N1	M0
IV	Any T	Any N	M1
Regional nodes (N)			
Nx	Regional lymph nodes cannot be assessed		
N0	No regional lymph node metastasis		
N1	Regional lymph node metastasis		
Distant metastasis (M)			
Mx	Distant metastasis cannot be assessed		
M0	No distant metastasis		
M1	Distant metastasis (excluding peritoneal metastasis)		

Notes

1. The primary site—that is, ovary, fallopian tube, or peritoneum—should be designated where possible. In some cases, it may not be possible to clearly delineate the primary site, and these should be listed as “undesignated”.

2. The histologic type should be recorded.

3. The staging includes a revision of the Stage III patients and allotment to Stage IIIA1 is based on spread to the retroperitoneal lymph nodes without intraperitoneal dissemination, because an analysis of these patients indicates that their survival is significantly better than those who have intraperitoneal dissemination.

4. Involvement of retroperitoneal lymph nodes must be proven cytologically or histologically.

5. Extension of tumor from omentum to spleen or liver (Stage IIIC) should be differentiated from isolated parenchymal splenic or liver metastases (Stage IVB).

^a^ Source: Prat J.^17^

In addition to these changes, several other modifications of the former staging system have been made to better prospectively capture the data. Stage IC is now divided into three categories: IC1 (surgical spill); IC2 (capsule ruptured before surgery or tumor on ovarian or fallopian tube surface); and IC3 (malignant cells in the ascites or peritoneal washings). Stage IIC has been eliminated. The updated staging includes a revision of the Stage IIIC based on spread to the retroperitoneal lymph nodes alone without intraperitoneal dissemination because an analysis of these patients indicates that their survival is significantly better than those who have intraperitoneal dissemination.^18^ This category is now subdivided into IIIA1(i) (metastasis ≤10 mm in greatest dimension), and IIIA1(ii) (metastasis >10 mm in greatest dimension). Stage IIIA2 is now “microscopic extrapelvic peritoneal involvement with or without positive retroperitoneal lymph node” metastasis. The wording of Stage IIIB has been modified to reflect the lymph node status. Stage IVB now includes metastases to the inguinal lymph nodes.

##### Regional lymph nodes (N)

- NX: Regional lymph nodes cannot be assessed.
- N0: No regional lymph node metastasis.
- N1: Regional lymph node metastasis.

##### Distant metastasis (M)

- MX: Distant metastasis cannot be assessed.
- M0: No distant metastasis.
- M1: Distant metastasis (excluding peritoneal metastasis).

### 1.3 Histopathologic classification

The majority of cases of ovarian cancer are of epithelial origin. FIGO endorses the WHO histologic typing of epithelial ovarian tumors. It is recommended that all ovarian epithelial tumors be subdivided according to the classification given below.^19^

The histologic classification of ovarian, fallopian tube, and peritoneal neoplasia is as follows:

- Serous tumors.
- Mucinous tumors.
- Endometrioid tumors.
- Clear cell tumors.
- Brenner tumors.
- Undifferentiated carcinomas (this group of malignant tumors is of epithelial structure, but they are too poorly differentiated to be placed in any other group).
- Mixed epithelial tumors (these tumors are composed of two or more of the five major cell types of common epithelial tumors. The types are usually specified).
- Cases with high‐grade serous carcinoma in which the ovaries and fallopian tubes appear to be incidentally involved and not the primary origin can be labeled as peritoneal carcinoma or serous carcinoma of undesignated site, at the discretion of the pathologist.

Epithelial tumors of the ovary and fallopian tube are further subclassified by histologic grading, which can be correlated with prognosis. This grading system does not apply to nonepithelial tumors.^20^ Two grading systems are applied. For nonserous carcinomas (most endometrioid and mucinous), grading is identical to that used in the uterus, based on architecture with a one‐step upgrade if there is prominent nuclear atypia, as follows:

- GX: Grade cannot be assessed.
- G1: Well differentiated.
- G2: Moderately differentiated.
- G3: Poorly differentiated.

Serous carcinomas are the most common in both the ovary and tube. More than 90% of fallopian tube carcinomas are serous or high‐grade endometrioid adenocarcinoma. Other cell types have been reported, but are rare.^1^, ^2^, ^21^ Serous carcinomas are graded in a two‐grade system befitting their biology. High‐grade serous carcinomas, including both classic appearing and those with SET features (solid, endometrioid‐like, and transitional) carry a high frequency of mutations in *TP53*.^22^, ^23^, ^24^ Low‐grade serous carcinomas are often associated with borderline or atypical proliferative serous tumors, often contain mutations in *BRAF* and *KRAS* and contain wild‐type *TP53*. Most “moderately differentiated” serous carcinomas carry mutations in *TP53* and should be combined with the high‐grade tumors.^20^, ^23^, ^24^, ^25^

Nonepithelial cancers, although uncommon, are extremely important. These include granulosa cell tumors, germ cell tumors, sarcomas, and lymphomas. They are discussed below as separate entities. Metastatic neoplasms to the ovary, such as tumors arising in the breast, lower reproductive tract sites (cervix or uterine carcinomas) and gastrointestinal tract (signet ring cell [Krukenberg] carcinomas, low grade appendiceal or pancreaticobiliary mucinous tumors and other neoplasms) are graded and staged in accordance with their respective sites of origin.^1^, ^2^

## 2 EPIDEMIOLOGY

Malignant tumors of the ovaries occur at all ages with variation in histologic subtype by age. For example, in women younger than 20 years of age, germ cell tumors predominate, while borderline tumors typically occur in women in their 30s and 40s—10 or more years younger than in women with invasive epithelial ovarian cancers, which mostly occur after the age of 50 years.

The lifetime risk of a woman in the USA developing ovarian cancer is approximately 1 in 70. Approximately 23% of gynecologic cancers are ovarian in origin, but 47% of all deaths from cancer of the female genital tract occur in women with ovarian cancer. Overall, epithelial ovarian cancer accounts for 4% of all new cancer diagnoses in women and 5% of all cancer‐related deaths.^1^, ^2^, ^26^

The overall incidence of epithelial tumors varies from 9–17 per 100 000 and is highest in high‐income countries, with the exception of Japan.^27^ However, this incidence rate increases proportionately with age. The largest number of patients with epithelial ovarian cancer is found in the 60–64 years age group. The median age is about a decade earlier in low‐income countries.

Established risk factors for epithelial ovarian tumors include reproductive risk factors. Women who have never had children are twice as likely to develop this disease. First pregnancy at an early age, early menopause, and the use of oral contraceptives have been associated with lower risks of ovarian cancer.^28^ The relationship of these variables to fallopian tube cancer is unclear.

As noted above, it has been previously presumed that fallopian tube malignancies were rare; however, this has been challenged by evidence to show that many tumors that were classified as serous carcinomas of the ovary or peritoneal cancers appear to have their origin in the fallopian tube.^3^, ^4^, ^5^, ^6^, ^7^ When the origin is uncertain, the convention of designating all serous cancers as originating in the ovary should no longer be used and the term “undesignated origin” may be applied at the discretion of the pathologist.^19^

### 2.1 Genetics

Hereditary factors are implicated in approximately 20% of ovarian, fallopian tube, and peritoneal cancers^29^, ^30^, ^31^, ^32^, ^33^:

1. Most hereditary ovarian cancers are due to pathogenic mutations in either the *BRCA1* or *BRCA2* genes. At least 15% of women with high‐grade nonmucinous ovarian cancers have germline mutations in *BRCA1*/*2* and, importantly, almost 40% of these women do not have a family history of breast/ovarian cancer. All women with high‐grade nonmucinous invasive ovarian cancers should be offered genetic testing even if they do not have a family history of breast/ovarian cancer.
2. Inherited deleterious mutations in *BRCA1* and *BRCA2* are the major genetic risk factors. Women who carry germline mutations in *BRCA1* and *BRCA2* have a substantially increased risk of ovarian, tubal, and peritoneal cancer—about 20%–50% with *BRCA1* and 10%–20% with *BRCA2*.^30^, ^31^, ^32^, ^33^ Typically, these cancers occur at an earlier age than sporadic cancers, particularly in *BRCA1* mutation carriers, with a median age of diagnosis in the mid‐40s.
3. A number of other low‐ to moderate‐penetrance genes can also predispose to ovarian, fallopian tube, or peritoneal cancer. A study using next generation sequencing of constitutional DNA samples from 1915 women with ovarian cancer was carried out to identify germline mutations using a panel of 20 genes including *BRCA1* and *BRCA2*, DNA mismatch repair genes, double‐stranded DNA break repair genes such as *CHEK2* and *ATM*, as well as the *BRCA1*‐associated complex or the *BRCA2*/Fanconi Anemia pathway genes (including *BRIP1*, *BARD1*, *PALB2*, *RAD50*, *RAD51C*, and *RAD51D*, among others). About 80% of mutations were in *BRCA1* or *BRCA2*. About 3% of patients carried mutations in the Fanconi Anemia pathway genes, while only 0.4% had mutations in mismatch repair genes.^34^ In an earlier similar study that included 360 patients, 24% carried germline loss‐of‐function mutations including 18% in *BRCA1* or *BRCA2* and 6% in *BARD1*, *BRIP1*, *CHEK2*, *MRE11A*, *MSH6*, *NBN*, *PALB2*, *RAD50*, *RAD51C*, or *TP53*.^35^, ^36^
4. Inherited mutations in the mismatch repair genes associated with Lynch syndrome type II. Women carrying these mutations have an increased risk of a number of cancers including colon, endometrial, and ovarian cancer. Typically, the ovarian cancers that occur are endometrioid or clear cell histologically and are usually Stage I.^36^

Women with a strong family history of epithelial ovarian, fallopian tube, or peritoneal cancers, particularly if there is a documented germline BRCA mutation, are advised to have a risk‐reducing bilateral salpingo‐oophorectomy after appropriate counseling and at the completion of childbearing. All women who are suspected of carrying a BRCA germline mutation, based on family history or young age of diagnosis and a high‐grade serous or high‐grade endometrioid cancer, should be offered genetic testing. BRCA mutations may also occur in women without a family history of breast/ovarian cancer, and genetic testing should be considered in patients from ethnic groups where there is a high incidence of founder mutations (e.g. Ashkenazi Jewish ancestry), and in women with high‐grade serous cancers under the age of 70 years. Australian guidelines advise that all women with invasive epithelial ovarian cancer apart from mucinous cancers diagnosed under the age of 70 should be offered BRCA mutation testing independent of family history and histologic subtype.^37^ In contrast, the Society of Gynecologic Oncology (SGO) and National Comprehensive Cancer Network (NCCN) guidelines recommend that all women diagnosed with ovarian, fallopian tube, or peritoneal carcinoma, regardless of age or family history, should receive genetic counseling and be offered genetic testing.^38^ Women whose family history suggests Lynch syndrome type II should undergo appropriate genetic counseling and testing.

## 3 SCREENING

To date, there are no documented effective screening methods that reduce the mortality of ovarian, fallopian tube, or peritoneal cancers. Studies using CA125, ultrasonography of the pelvis, and pelvic examination do not have an acceptable level of sensitivity and specificity, based on trials carried out in women in the general population^39^, ^40^ and those in the high‐risk population.^41^, ^42^ The US Preventive Services Task Force recommends against screening asymptomatic women for ovarian cancer with pelvic examination, pelvic ultrasound, or serum tumor marker measurements.^43^ The low prevalence of disease and lack of high‐quality screening methods make it more likely to obtain false‐positive results leading to unnecessary interventions. A recent study of multimodal screening using CA125 based on a risk of ovarian cancer algorithm (ROCA) every 4 months and transvaginal ultrasound annually or earlier where indicated by the ROCA in women at high risk of ovarian cancer reported that screening was associated with a low rate of high‐volume disease at primary surgery and very high rates of no residual disease after surgery.^44^ Given that the majority of women with advanced stage ovarian cancer, even with complete resection, will relapse after chemotherapy, this does not seem to be a good alternative to risk‐reducing surgery. The authors of the screening study concluded that risk‐reducing salpingectomy‐oophorectomy remains the treatment choice for women at high risk of ovarian/fallopian tube cancer.^44^

Women at increased genetic risk should be encouraged to consider risk‐reducing bilateral salpingo‐oophorectomy, as this is the most effective way to reduce mortality in this population of women.^40^, ^41^ A bulletin from the American College of Obstetricians and Gynecologists (ACOG) has recommended that opportunistic (at the time of a clinically indicated hysterectomy) bilateral salpingectomy be considered in women not at genetic risk who wish to retain their ovaries as a way to reduce their risk of later developing high‐grade serous carcinomas.^45^

## 4 DIAGNOSIS

Patients with epithelial ovarian cancers confined to the ovary or fallopian tube at initial diagnosis have a very good prognosis.^46^, ^47^, ^48^, ^49^ The symptoms are often very insidious and the duration of symptoms not very different between patients with early stage or advanced stage disease.^13^, ^14^ This may reflect the different biological behavior of the various histologic subtypes; for example, grade 1 serous, clear cell, mucinous, and endometrioid cancers are commonly early stage at presentation, whereas high‐grade serous cancers are most often Stage III because of early dissemination by a more aggressive cancer. Tumor markers such as human gonadotropin (hCG) and alpha‐fetoprotein (AFP) are mandatory to exclude germ cell tumors in younger patients with a pelvic mass or suspicious enlargement of an ovary.

Approximately two‐thirds of all epithelial “ovarian” cancers are Stage III or Stage IV at diagnosis. Presenting symptoms include vague abdominal pain or discomfort, menstrual irregularities, dyspepsia, and other mild digestive disturbances, which may have been present for only a few weeks.^13^, ^14^, ^50^ As the disease progresses, abdominal distention and discomfort from ascites generally worsen, and may be associated with respiratory symptoms from increased intra‐abdominal pressure or from the transudation of fluid into the pleural cavities. Abnormal vaginal bleeding is an uncommon symptom.

Serous fallopian tube and peritoneal cancers present the same as ovarian cancer. Past analyses have been biased because many fallopian tube cancers have been presumed to arise in the ovaries.

A detailed medical history must be taken to ascertain possible risk factors, history of other cancers, and history of cancer in the family. Then a complete physical examination, including general, breast, pelvic, and rectal examination, must be performed.^1^

Prior to surgery a chest radiograph should be taken to screen for a pleural effusion and a CT scan of the abdomen and pelvis should be performed to delineate the extent of intra‐abdominal disease. However, in the absence of extra‐abdominopelvic disease, radiological scanning does not replace surgical staging with laparotomy. Tumor markers including CA125, and carcinoembryonic antigen (CEA) should be considered.^1^ With a high CA125 level, the most common diagnosis would be epithelial ovarian, fallopian tube, or peritoneal cancer.

A gastric or colonic primary with metastases to the ovaries may mimic ovarian cancer, and if CEA or CA19‐9 are elevated, this should be considered. A ratio of more than 25:1 (CA125 and CEA) favors an ovarian primary although it does not completely rule out a primary in the gastrointestinal tract.^51^

A current mammogram should be considered as patients are frequently in the age group where breast cancer is prevalent. A colonoscopy is indicated when symptoms suggest possible colorectal cancer.^1^

The following factors point to the presence of a malignancy, and are useful in the clinical assessment of masses:

- Age of the patient (young for germ cell, older for epithelial malignancies).
- Bilaterality.
- Tumor fixation clinically.
- Ascites.
- Ultrasonographically complex, especially if solid areas.
- CT finding of metastatic nodules.
- Elevated tumor markers.

## 5 PRIMARY SURGERY

In general, the prognosis of epithelial ovarian, fallopian, and peritoneal malignancies is independently affected by the following^1^, ^52^, ^53^:

- Stage of the cancer at diagnosis.
- Histologic type and grade.
- Maximum diameter of residual disease after cytoreductive surgery.

### 5.1 Staging laparotomy

A thorough staging laparotomy is an important part of early management. If the preoperative suspicion is malignancy, a laparotomy should be performed. If there is no visible or palpable evidence of metastasis, the following should be performed for adequate staging^1^, ^10^, ^11^:

- Careful evaluation of all peritoneal surfaces.
- Retrieval of any peritoneal fluid or ascites. If there is none, washings of the peritoneal cavity should be performed.
- Infracolic omentectomy.
- Selective lymphadenectomy of the pelvic and para‐aortic lymph nodes, at least ipsilateral if the malignancy is unilateral.
- Biopsy or resection of any suspicious lesions, masses, or adhesions.
- Random peritoneal biopsies of normal surfaces, including from the undersurface of the right hemidiaphragm, bladder reflection, cul‐de‐sac, right and left paracolic recesses, and both pelvic sidewalls.
- Total abdominal hysterectomy and bilateral salpingo‐oophorectomy in most cases.
- Appendectomy for mucinous tumors if the appendix appears abnormal.

Upon opening the abdominopelvic cavity, the peritoneal fluid should be sent for cytology. In the absence of ascites, irrigation should be performed and washings sent for cytology.

The laparotomy should proceed with a detailed examination of the contents, including all of the peritoneal surfaces. In addition to the suspicious sites, biopsies from the peritoneal reflection of the bladder, the posterior cul‐de‐sac, both paracolic gutters, subdiaphragmatic surfaces, and both pelvic sidewalls should be taken. The primary tumor, if limited to the ovary, should be examined to look for capsular rupture. All obvious sites of tumor must be removed wherever possible in addition to total hysterectomy and bilateral salpingo‐oophorectomy. The omentum, pelvic, and para‐aortic lymph nodes should be removed for histologic examination.

In younger women, fertility preservation may be desired. In these patients, conservative surgery, with preservation of the uterus and contralateral ovary, should be considered after informed consent.^47^

Clinical judgment is important in the approach to a pelvic mass in the young, reproductive‐aged woman. If the suspicion is strong for malignancy, open laparotomy is generally indicated. Laparoscopy may be more appropriate if the suspicion is more for benign disease, where tumor markers (including hCG and AFP) are normal. A biopsy of any suspicious lesion can be performed and frozen section obtained in order to proceed expeditiously with definitive surgery.

Ovaries and fallopian tubes should be evaluated as thoroughly as possible to establish the site of origin. If visible, the entire tube, particularly the distal portion, should be submitted for pathology and examined using the SEE‐FIM protocol.^33^ Ovaries should be scrutinized for coexisting endometriotic cysts, adenofibromas, or other benign conditions that could serve as a nidus of tumor development.

### 5.2 Cytoreductive (debulking) surgery for advanced stage disease

#### 5.2.1 Primary debulking

At least two‐thirds of patients with ovarian cancer present with Stage III or IV disease. This may affect the performance status and fitness for surgery. However, the most important prognostic indicator in patients with advanced stage ovarian cancer is the volume of residual disease after surgical debulking. Therefore, patients whose medical condition permits should generally undergo a primary laparotomy with total abdominal hysterectomy, bilateral salpingo‐oophorectomy, omentectomy, and maximal attempt at optimal cytoreduction.^1^, ^52^, ^53^ This may necessitate bowel resection, and occasionally, partial or complete resection of other organs. Based on recent data from the randomized Lymphadenectomy in Ovarian Neoplasm (LION) trial, the removal of clinically negative lymph nodes during cytoreductive surgery does not increase the progression‐free or overall survival and should not be undertaken.^54^ **Level of Evidence A**.

#### 5.2.2 Interval debulking

In selected patients with cytologically proven Stage IIIC and IV disease who may not be good surgical candidates, 3–4 cycles of neoadjuvant chemotherapy (NACT) may be given initially, followed by interval debulking surgery and additional chemotherapy as demonstrated in the EROTC and CHORUS Trials.^55^, ^56^ These two randomized prospective trials showed that in selected patients, interval debulking surgery after neoadjuvant chemotherapy showed equivalent survival with less morbidity compared with primary cytoreductive surgery. NACT followed by interval debulking surgery may be particularly useful in patients with a poor performance status, significant medical comorbidities, visceral metastases, and those who have large pleural effusions and/or gross ascites.^57^, ^58^ In selected patients whose primary cytoreduction is considered suboptimal, particularly if a gynecologic oncologist did not perform the initial operation, interval debulking may be considered after 2–3 cycles of systemic chemotherapy.^1^, ^55^, ^56^, ^59^ Pathologic assessment for residual tumor following neoadjuvant therapy will enable an estimate of residual disease and pathological response.^60^ There are recent data to indicate that patients who have a good pathological response have a better outcome. A histopathologic scoring system for measuring response to neoadjuvant chemotherapy has been developed and validated by Bohm et al.^61^ who reported criteria for defining a chemotherapy response score (CRS) based on a three‐tier system. A CRS 3 (complete or near complete pathological response) was associated with a better prognosis. Recently, these results have been validated in an independent West Australian cohort.^62^

## 6 CHEMOTHERAPY

### 6.1 Chemotherapy for early‐stage cancer

The prognosis of patients with adequately staged tumors with Stage IA and Stage IB grade 1–2 epithelial cancers of the ovary is very good; adjuvant chemotherapy does not provide additional benefits and is not indicated. For higher‐grade tumors and for patients with Stage IC disease, adjuvant platinum‐based chemotherapy is given to most patients, although there has been debate about the absolute survival benefit in women with Stage IA and IB cancers who have had thorough surgical staging.^46^ All patients with Stage II disease should receive adjuvant chemotherapy. The optimal number of cycles in patients with Stage I disease has not been definitively established, but typically between 3 and 6 cycles are administered. The Gynecologic Oncology Group (GOG) 157 study suggested that 3 cycles of carboplatin and paclitaxel was equivalent to 6 cycles,^49^ but in subgroup analysis, 6 cycles appeared superior in patients with high‐grade serous cancers.^63^

There is no evidence to support adjuvant therapy for carcinoma in situ of the fallopian tube and it is not recommended.^1^, ^2^, ^64^ **Level of Evidence A**.

### 6.2 Chemotherapy for advanced stage ovarian cancer

Patients who have had primary cytoreduction should receive chemotherapy following surgery^1^, ^65^ (Table 3). The accepted standard is 6 cycles of platinum‐based combination chemotherapy, with a platinum (carboplatin or cisplatin) and a taxane (paclitaxel or docetaxel).^66^, ^67^, ^68^, ^69^, ^70^ Docetaxel is an option in patients who have had a significant allergic reaction to paclitaxel or who develop early sensory neuropathy as it has less neurotoxicity, but it is more myelosuppressive than paclitaxel. The SCOT‐ROC (Scottish Gynecological Cancer Trials Group) study randomly assigned 1077 women with Stages IC–IV epithelial ovarian cancer to carboplatin paclitaxel or docetaxel.^71^ The efficacy of docetaxel was similar to paclitaxel. The median progression‐free survival was 15.1 months versus 15.4 months. The MITO‐2 trial randomized over 800 patients to receive either carboplatin and liposomal doxorubicin (PLD) or carboplatin and paclitaxel. The median progression‐free survival was 19.0 months and 16.8 months with carboplatin/PLD and carboplatin/paclitaxel, respectively.^72^ The median overall survival times were 61.6 months and 53.2 months with carboplatin/PLD and carboplatin/paclitaxel, respectively (hazard ratio [HR] 0.89; 95% CI, 0.72–1.12; *P* = 0.32). Carboplatin/PLD produced a similar response rate but different toxicity (less neurotoxicity and alopecia but more hematologic adverse effects) and could also be considered as an option in patients where paclitaxel cannot be used.

**TABLE 3:** Chemotherapy for advanced epithelial ovarian cancer: recommended regimens^a^

Drugs Standard regimens	Dose	Administration (h)	Interval	No. of treatments
Carboplatin	AUC =5–6	3	Every 3 weeks	6–8 cycles
Paclitaxel	175 mg/m^2^			
Carboplatin	AUC =5–6	3	Every 3 weeks	6 cycles
Paclitaxel	80 mg/m^2^		Every week	18 weeks
Carboplatin	AUC =5	3	Every week	6 cycles
Docetaxel	75 mg/m^2^		Every 3 weeks	
Cisplatin	75 mg/m^2^	3	Every 3 weeks	6 cycles
Paclitaxel	135 mg/m^2^			
Carboplatin (single agent)^b^	AUC =5	3	Every 3 weeks	6 cycles, as tolerated

Abbreviation: AUC, area under the curve dose by the methods of Calvert et al.^73^, ^74^

^a^ Reproduced with permission from Berek et al.^1^

^b^ In patients who are elderly, frail, or poor performance status.

Although intraperitoneal chemotherapy has been shown to be associated with improved progression‐free survival and overall survival in selected patients with optimally debulked Stage III ovarian cancer, it is not widely used outside the USA because of concerns regarding increased toxicity and catheter‐related problems, and the benefits are still debated.^75^, ^76^, ^77^, ^78^ The GOG 172 trial compared intravenous paclitaxel plus cisplatin with intravenous paclitaxel plus intraperitoneal cisplatin and paclitaxel in patients with Stage III ovarian or peritoneal carcinoma, with no residual disease greater than 1 cm in diameter.^77^ Only 42% of patients in the intraperitoneal group completed 6 cycles of the assigned therapy, but the intraperitoneal group had an improvement in progression‐free survival of 5.5 months (23.8 months vs 18.3 months; *P* = 0.05) and an improvement in overall survival of 15.9 months (65.6 months vs 49.7 months; *P* = 0.03). **Level of Evidence A**.

More recently, the GOG 252 trial reported a median progression‐free survival of approximately 27–29 months in over 1500 patients with optimal Stage II–III disease treated with regimens consisting of different combinations of intravenous and intraperitoneal cisplatin, carboplatin, and paclitaxel, in combination with bevacizumab, which raises questions about the role of intraperitoneal chemotherapy.^79^ The treatment arms included intravenous carboplatin AUC 6/intravenous weekly paclitaxel at 80 mg/m^2^; intraperitoneal carboplatin AUC 6/intravenous weekly paclitaxel at 80 mg/m; and intravenous paclitaxel at 135 mg/m^2^ on day one/intraperitoneal cisplatin at 75 mg/m^2^ on day two/intraperitoneal paclitaxel at 60 mg/m^2^ on day eight. In addition, each arm received intravenous bevacizumab at 15 mg/kg with cycles 2 through 6 of chemotherapy and then alone for cycles 7 through 22. The median progression‐free survival by intent‐to‐treat analysis was 24.9 months (intravenous carboplatin), 27.3 months (intraperitoneal carboplatin), and 26.0 months (intraperitoneal cisplatin). An analysis limited to patients with optimal Stage III tumors and no gross residual disease found a median progression‐free survival of 31–34 months in all three arms. The median overall survival for all patients enrolled was 75.5 months, 78.9 months, and 72.9 months, respectively, and median overall survival for Stage II/III with no gross residual disease was 98.8 months, 104.8 months, and not reached.^79^ By comparison, the GOG 172 trial comparing intraperitoneal and intravenous chemotherapy regimens in ovarian cancer had a median progression‐free survival of 23.8 months with intraperitoneal cisplatin (vs 18.3 months with intravenous) with an improvement in overall survival in favor of intraperitoneal injection.^77^ In addition, the median progression‐free survival was 60 months in the patients with no residual disease in GOG 172. Differences in the cisplatin arm from the GOG 172 study include a dose reduction from 100 mg to 75 mg and a shorter infusion time from 24 to 3 h.^77^ If intraperitoneal treatment is used it would be appropriate to follow the GOG 172 protocol rather than the modified protocol with a lower dose of cisplatin accepting the increased toxicity.

Combination chemotherapy with either intravenous carboplatin and paclitaxel or intraperitoneal cisplatin and paclitaxel (using the GOG 172 protocol) are the standard treatment options for patients with advanced disease, with evidence to support the addition of bevacizumab as well. The advantages and disadvantages of the intravenous versus intraperitoneal routes of administration of these drugs should be discussed with the patient in light of the results of GOG 252 discussed above, which did not demonstrate improved outcomes with intraperitoneal chemotherapy when bevacizumab was added to intravenous chemotherapy. Intraperitoneal chemotherapy is applicable only to patients with advanced disease who have had optimal debulking and have less than 1 cm residual disease. It should be used only in centers that have experience with intraperitoneal chemotherapy.

The recommended doses and schedule for intravenous chemotherapy are: carboplatin (starting dose AUC 5–6), and paclitaxel (175 mg/m^2^), every 3 weeks for 6 cycles.^1^

The Japanese GOG (JGOG) reported an alternative dose‐dense regimen of carboplatin AUC 6 every 3 weeks for 6 cycles and weekly paclitaxel 80 mg/m^2^ and showed improved progression‐free survival and overall survival.^80^, ^81^ An Italian trial (MITO‐7) investigated a different schedule of weekly carboplatin (AUC 2 mg/mL per min) plus weekly paclitaxel (60 mg/m^2^) compared with carboplatin (AUC 6 mg/mL per min, administered every 3 weeks) and paclitaxel (175 mg/m^2^).^82^ The co‐primary endpoints were progression‐free survival and quality of life, which is quite unique for an ovarian cancer trial. The weekly regimen did not significantly improve progression‐free survival compared with the conventional regimen (18.8 months vs 16.5 months; *P* = 0.18), but was associated with better quality of life and fewer toxic effects and could be considered a reasonable option, particularly in elderly patients in whom combination chemotherapy is planned. The results of the ICON8 trial investigating dose‐dense paclitaxel in a non‐Japanese population have been recently reported.^83^ Over 1500 predominantly white patients were randomized to receive one of three regimens: Arm 1: carboplatin AUC 5/6 and paclitaxel 175 mg/m^2^ every 3 weeks; Arm 2: carboplatin AUC 5/6 every 3 weeks and paclitaxel 80 mg/m^2^ weekly; and Arm 3: carboplatin AUC 2 and paclitaxel 80 mg/m^2^ weekly. All patients had received neoadjuvant chemotherapy with planned interval debulking or received chemotherapy after initial primary cytoreductive surgery. There was no benefit found for the dose‐dense regimens. The progression‐free survival was 24.4 months with every 3‐week dosing, compared with 24.9 months and 25.3 months in arms 2 and 3, respectively. The overall survival was reported recently and the median overall survival was 47.4 months, 54.1 months, and 53.4 months in arms 1, 2, and 3, respectively.^84^ These results are very different to the JGOG trial and it seems that the likely explanation is due to pharmacogenomic differences between these two ethnic groups.^85^

The recommended doses and schedule for intraperitoneal chemotherapy are paclitaxel 135 mg/m^2^ intravenously on day one, followed by cisplatin 100 mg/m^2^ intraperitoneally on day two, followed by paclitaxel 60 mg/m^2^ intraperitoneally on day eight, every 3 weeks for 6 cycles, as tolerated.^77^, ^78^ Many centers modify the dose of cisplatin to 75 mg/m^2^ rather than 100 mg/m^2^ that was used in GOG 172 to reduce toxicity, but this could be questioned based on GOG 252 results discussed above. Others substitute carboplatin (AUC 5–6) for cisplatin in the regimen and the same caveats regarding lack of evidence apply. The role of intraperitoneal carboplatin is being evaluated in the JGOG iPocc trial, and the results should be available in the near future.

Bevacizumab 7.5–15 mg/kg every 3 weeks may be added to these regimens. Two studies (GOG 218 and ICON7) have reported a modest, but statistically significant increase in progression‐free survival in patients receiving maintenance bevacizumab following carboplatin, paclitaxel, and concurrent bevacizumab.^86^, ^87^ The GOG 218 trial randomized patients with Stage III and macroscopic residual disease as well as Stage IV ovarian cancer to: (1) 6 cycles of carboplatin and paclitaxel plus placebo for cycles 2 through 22 (control group); (2) 6 cycles of carboplatin and paclitaxel in combination with bevacizumab (15 mg/kg) for cycles 2 through 6, followed by placebo (initiation group); and (3) 6 cycles of carboplatin and paclitaxel with bevacizumab for cycles 2 through 22 (throughout group). The median progression‐free survival was 10.3 months versus 11.2 months versus 14.1 months in control versus initiation versus throughout group.^87^ The ICON7 trial included patients with early‐stage high‐risk disease (Stage I or IIA clear cell or grade 3) and advanced Stage IIB–IV and randomized to 6 cycles of chemotherapy or 6 cycles of chemotherapy plus bevacizumab (7.5 mg/kg), followed by 12 cycles of maintenance bevacizumab.^86^ Restricted mean progression‐free survival was statistically different with 22.4 months versus 24.1 months (control vs bevacizumab) although the clinical significance can be questioned. There is no evidence to demonstrate an overall survival benefit, but a subgroup analysis of the ICON7 trial reported an improved median survival (30.3 months vs 39.4 months) in patients with suboptimal Stage III and Stage IV.^86^, ^88^ The role, optimal dose (7.5 mg/kg vs 15 mg/kg), timing (primary vs recurrent disease), and duration of treatment of bevacizumab are still debatable. Similarly, there was no difference in overall survival between the three arms in GOG 218, but in an exploratory subgroup analysis the median overall survival for Stage IV disease was 32.6 months versus 42.8 months (control vs throughout).^89^

van Driel et al.^90^ reported results of a randomized trial in which 245 patients with Stage III epithelial ovarian cancer who had received 3 cycles of neoadjuvant chemotherapy underwent interval debulking surgery. These patients were then randomized to receive either 3 more cycles of paclitaxel plus carboplatin with or without hyperthermic intraperitoneal chemotherapy (HIPEC). The addition of HIPEC to interval cytoreductive surgery resulted in longer recurrence‐free survival (14.2 months vs 10.7 months) and overall survival (45.7 months vs 33.9 months) and did not result in higher rates of adverse effects. These findings are provocative and raise important questions. Unfortunately, the study did not have an arm with intraperitoneal cisplatin alone without HIPEC, therefore it is not possible to know whether the improved survival was due to the addition of intraperitoneal cisplatin alone or HIPEC. Confirmatory trials are in progress to determine the role of HIPEC.

In patients who may not tolerate combination chemotherapy because of medical comorbidities, frailty, or advanced age, single‐agent, intravenously administered carboplatin (AUC 5–6) can be given. However, this approach has been challenged by the EWOC‐1 trial,^91^ a randomized phase 2 trial that enrolled 120 vulnerable and elderly patients to either carboplatin (AUC 5) and paclitaxel 175 mg/m^2^ every 3 weeks for 6 cycles (Arm A), carboplatin (AUC 5–6) alone every 3 weeks for 6 cycles (Arm B), or weekly carboplatin (AUC 2) and paclitaxel 60 mg/m^2^ weekly for 18 weeks (Arm C). The median progression‐free survival was 12.5 months (95% CI, 10.3–15.3), 4.8 months (95% CI, 3.8–15.3), and 8.3 months (95% CI, 6.6–15.3), respectively (*P* < 0.001), and median overall survival for arm A–B–C was not reached (NR) (21, NR), 7.4 (5.3–NR), and 17.3 (10.8–NR), respectively (*P* = 0.001). The Independent Data Monitoring Committee (IDMC) recommended that the study be closed as survival in arm B (carboplatin alone) was significantly worse than the combination arms. The findings of this trial raise questions about the place of single‐agent carboplatin, but it should be noted that it was a small trial and the findings need to be confirmed.

### 6.3 Neoadjuvant chemotherapy

An increasing proportion of patients with advanced stage ovarian cancer are being treated with upfront neoadjuvant chemotherapy (NACT) for 3–4 cycles prior to interval debulking and further chemotherapy. This is based on the results of four trials that have reported equivalent outcomes for progression‐free survival and overall survival, but less morbidity and lower mortality compared with primary debulking surgery (PDS).^57^ Vergote et al.^55^ reported the results in 2010 of the first randomized EORTC‐NCIC (National Cancer Institute of Canada) study of PDS versus three cycles of NACT followed by interval debulking. All the patients had extensive Stage IIIC or IV disease. Patients were randomly assigned either to PDS followed by at least six courses of platinum‐based chemotherapy or to three cycles of neoadjuvant platinum‐based chemotherapy followed by interval debulking surgery in all patients with a response or stable disease, followed by at least three further courses of platinum‐based chemotherapy. The median progression‐free survival in both groups was 12 months. The median overall survival was also similar at 29 months versus 30 months (PDS vs NACT). There was lower postoperative morbidity and mortality in the NACT group. The median overall survival was considerably less than the 60+ months expected with PDS and optimal cytoreduction followed by chemotherapy, suggesting that the study included a cohort of patients with very advanced disease and poor prognosis. The study provoked much discussion and debate regarding the role of NACT.

The Chemotherapy or Upfront Surgery (CHORUS) trial randomized patients to NACT followed by interval debulking and then three additional cycles or PDS followed by six cycles of platinum‐based chemotherapy.^56^ The optimal debulking rate was only 16% in the PDS group compared with 40% following NACT, which are lower than would be expected. The median duration of surgery was only 120 min in both groups, which was criticized as it did not seem to be long enough for aggressive debulking surgery and optimal cytoreduction. There was a 5.6% postoperative mortality rate in the PDS group, which is high. The median progression‐free survival was 12 months in both groups, and the median overall survival was similar at 22.6 months versus 24.1 months (PDS vs NACT).

More recently the Japanese Oncology Group (JGOG 0602) reported the results of a randomized trial of NACT versus PDS in selected patients with Stage III–IV ovarian cancer.^92^ The primary endpoint was overall survival, and it was designed as a noninferiority trial. Between 2006 and 2011, 301 patients were randomized: 149 to PDS and 152 to NACT. The median overall survival was 49.0 months and 44.3 months in the PDS and NACT arms respectively. The hazard ratio (HR) for NACT was 1.052 (90.8% CI, 0.835–1.326), and one‐sided noninferiority *P* value was 0.24. In contrast to the previous two trials, the noninferiority of NACT was not confirmed with the caveat that this was a relatively small trial. The authors concluded that the noninferiority of NACT was not confirmed and that NACT may not always be a substitute for PDS.

The SCORPION trial investigated whether NACT followed by interval debulking surgery was superior to PDS in terms of perioperative complications and progression‐free survival in patients with advanced epithelial ovarian, fallopian tube, or primary peritoneal cancer with high tumor burden. Patients underwent initial laparoscopy to confirm Stage III/IV and to assess suitability for inclusion in the trial.^93^ They were randomized on the operating table to either immediate surgery or NACT. Of the 171 patients included, 84 were randomized to surgery and 87 to chemotherapy. They achieved a 47% complete resection rate with PDS compared with 77% in the NACT group and both arms achieved over 90% optimal resection. The aim was to demonstrate superiority of NACT over PDS, but the median progression‐free survival and overall survival were 14 months and 43 months for PDS and 14 months and 43 months for NACT. Consistent with other studies, the morbidity was greater in the PDS group with major complications occurring in 46% of the patients compared with 9.5% in the NACT group. Of concern, 8.3% of the PDS group died from surgical complications, while there were no postsurgical deaths in the NACT group. Hospital stays were significantly less for NACT.

It should be noted that both JGOG 0602 and SCORPION were carried out in expert centers selected for the skill and surgical expertise, but were both underpowered to demonstrate superiority or noninferiority of NACT versus PDS.

A recent systematic review and meta‐analysis that included four phase 3 trials with a total of 1692 patients concluded that NACT with carboplatin and paclitaxel followed by interval debulking surgery does not negatively impact the survival of women with advanced ovarian cancer compared with PDS, but that perioperative complications and mortality are significantly reduced by 70%–80%.^94^ Despite these four trials, there remain divergent views regarding the role of NACT. For selected patients with poor prognostic features, NACT seems advisable given equivalent outcomes for progression‐free survival and overall survival and lower perioperative morbidity and mortality. NACT is indicated in patients who are medically unfit for upfront surgery or who have a high risk of surgical morbidity and mortality, including those with parenchymal liver and lung metastases. However, PDS should be offered to patients with a good performance status and a more favorable prognosis. There are a number of models from the Mayo Clinic as well as the Memorial Sloan Kettering Cancer Center, among others, that have been advocated to improve patient selection for PDS as well as algorithms to guide management.

### 6.4 Maintenance chemotherapy

Almost 80% of women with advanced stage disease who respond to first‐line chemotherapy relapse. There have been several trials conducted to determine if there is a benefit of maintenance chemotherapy in these patients immediately following their primary treatment in an effort to decrease the relapse rate.^95^ These were all negative and there is no evidence to support maintenance chemotherapy after completion of first‐line therapy.

### 6.5 Maintenance therapy with PARP inhibitors

There is increasing evidence to support the role of maintenance therapy with PARP inhibitors following response to treatment in the first‐line therapy setting as well as in patients with platinum‐sensitive recurrent ovarian cancer. In the SOLO1 trial, patients with Stage III and IV high‐grade serous/high‐grade endometrioid ovarian cancer, a germline or somatic *BRCA1* or *2* mutation, and at least partial response to adjuvant platinum‐based chemotherapy were randomized to olaparib maintenance or placebo.^96^ A 70% risk reduction for progression of disease or death was seen for olaparib (HR 0.3) with a median progression‐free survival not reached versus 13.8 months with placebo. Twice as many patients were progression free after 3 years (60.4% versus 26.9%), which is unprecedented. More recently, 5‐year follow‐up data have been reported; at 5 years, 48% of patients randomized to 2 years of olaparib were progression free compared with 21% in the placebo arm. The median progression‐free survival was 56 months versus 13.8 months (HR 0.33).

The PRIMA trial enrolled a subset of patients considered to be at high risk of relapse and included patients with Stage III and IV high‐grade serous and endometrioid ovarian cancer with response to chemotherapy, regardless of BRCA status, and included those with suboptimal residual disease for Stage III after surgery as well as patients who received NACT and all patients with Stage IV.^97^ Patients were randomized to niraparib or placebo for 3 years. In the overall population, the median progression‐free survival was 8.2 months versus 13.8 months (control vs niraparib). In the homologous recombination deficient (HRD) subgroup as determined by the Myriad myChoice test (Myriad Genetics Inc, Salt Lake City, USA), the median progression‐free survival was 10.9 months versus 22.1 months. In the homologous recombination proficient subgroup, the difference was smaller although statistically significant (5.4 months vs 8.1 months).

In the PAOLA trial, patients with Stage III–IV high‐grade serous cancers regardless of BRCA status and at least partial response were randomized to bevacizumab or bevacizumab plus olaparib maintenance therapy.^98^ The median progression‐free survival for the intention‐to‐treat population was 16.6 months versus 22.1 months (without vs with olaparib); in the *BRCA*‐mutated group, median progression‐free survival was 21.7 months versus 37.2 months and in the HRD group excluding *BRCA*, median progression‐free survival was 16.6 months versus 28.1 months. However, in the HRD‐negative or unknown group the median progression‐free survival showed no difference (16 months vs 16.9 months). The PAOLA design did not include olaparib monotherapy, making it difficult to ascertain the contribution of bevacizumab.

The VELIA trial randomized patients with advanced stage ovarian cancer to: (1) platinum and paclitaxel chemotherapy (control); (2) veliparib with chemotherapy, and (3) veliparib with chemotherapy followed by veliparib maintenance.^99^ There was significant benefit from adding veliparib to chemotherapy and maintenance. In the BRCA mutation group, the median progression‐free survival was 22 months versus 34.7 months; in the HRD group, 20.5 months versus 31.9 months; and in the intention‐to‐treat population, 17.3 months versus 23.5 months. The results of the HR proficient patients were not reported.

All PARP inhibitors are associated with mainly low‐grade adverse effects, such as nausea, fatigue, and myelosuppression (anemia can be caused by all, neutropenia and thrombocytopenia mainly by niraparib), which can mostly be managed with dose reductions and interruptions.

There is also good evidence to support the role of PARP inhibitors as maintenance therapy following response to chemotherapy in patients with recurrent platinum‐sensitive ovarian cancer, as well as monotherapy in selected patients with recurrent ovarian cancer.^100^, ^101^, ^102^, ^103^, ^104^ Patients with BRCA mutations (both germline and somatic) have the greatest benefit, but a subset of patients with tumors with homologous recombination deficiency (HRD) also derive benefit from treatment with PARP inhibitors; the ongoing challenge is how best to identify these patients. The results of these trials are summarized in Table 4. Readers are directed to the article on targeted therapy by Basu et al.^105^ for further discussion of PARP inhibitors.

**TABLE 4:** Progression‐free survival endpoint in the three phase trials of maintenance PARP inhibitors

Study	PARP inhibitor progression‐free survival (months)	Placebo progression‐free survival (months)	Hazard ratio
**SOLO 2** ^102^	19.1	5.5	0.3
**NOVA** ^103^			
gBRCA	21	5.5	0.27
Non‐BRCA	9.3	3.9	0.45
Non‐BRCA HRD+	12.9	3.8	0.38
**ARIEL 3** ^104^			
gBRCA	16.6	5.4	0.23
HRD+ (includes WT/gBRCA)	13.6	5.4	0.32

### 6.6 Immune checkpoint inhibitors

There may be a potential role for immune checkpoint inhibitors in the first‐line therapy setting in combination with chemotherapy as well as in maintenance, either alone or in combination with a PARP inhibitor or angiogenesis inhibitor. A number of trials are addressing these important questions and the results are awaited. Unfortunately JAVELIN100, the first trial to be reported, was a negative trial.^106^ This was a randomized, open‐label, phase 3 trial that evaluated avelumab in combination with and/or following chemotherapy versus chemotherapy alone in 998 patients with previously untreated epithelial ovarian cancer. Progression‐free survival was not improved versus control, prespecified futility boundaries were crossed, and the trial was stopped. Time will tell whether there is a role for immune checkpoint inhibitors in the first‐line treatment of patients with ovarian cancer or whether it is possible to identify a subset who are most likely to derive benefit.

## 7 SECONDARY SURGERY

### 7.1 Second‐look laparotomy

A second‐look laparotomy (or laparoscopy) was previously performed in patients who have no clinical evidence of disease after completion of first‐line chemotherapy to determine response to treatment. Although of prognostic value, it has not been shown to influence survival, and is no longer recommended as part of the standard of care.^107^ **Level of Evidence C**.

### 7.2 Secondary cytoreduction

Secondary cytoreduction is defined as an attempt at cytoreductive surgery at some stage following completion of first‐line chemotherapy. Retrospective studies suggest that patients benefit if all macroscopic disease can be removed, which usually means patients with a solitary recurrence. Patients with a disease‐free interval longer than 12–24 months and those with only 1–2 sites of disease appear to derive most benefit.^108^, ^109^ The role of secondary cytoreductive surgery is being evaluated in randomized clinical trials. The role of secondary debulking surgery has been addressed in the DESKTOP III trial and the results recently presented on behalf of the AGO.^110^ This study included patients with a progression‐free survival of greater than 6 months after first‐line chemotherapy and who were considered to be good candidates for surgery based on a positive AGO Study Group score, defined as an ECOG performance status score of zero, ascites of 500 mL or less, and complete resection at initial surgery. Du Bois et al.^110^ reported that the median progression‐free survival in 204 women who met these criteria and who were randomized to undergo surgery followed by chemotherapy was 18.4 months, compared with 14 months in 203 women who were randomized to receive only second‐line chemotherapy. Median overall survival showed an overall survival benefit of more than 12 months for patients undergoing complete secondary cytoreduction (60.7 months vs 46.2 months). Overall survival for patients who underwent surgery and were only incompletely cytoreduced was only 28 months, stressing the importance of complete cytoreduction. Results of the GOG 213 trial, however, showed no statistically significant difference in progression‐free survival of 18.9 months versus 16.2 months, and overall survival of 50.6 months versus 64.7 months (with vs without secondary cytoreduction).^111^ In the view of these two trials, secondary cytoreduction can be considered a safe option for carefully selected patients. **Level of Evidence B**.

## 8 FOLLOW‐UP FOR MALIGNANT EPITHELIAL TUMORS

There is no evidence to show that intensive clinical monitoring during follow‐up after completion of primary surgery and chemotherapy with early initiation of chemotherapy in asymptomatic women with recurrent disease improves overall survival or quality of life. In asymptomatic patients with CA125 progression and small volume disease or no radiological evidence of recurrence, it is appropriate to delay starting chemotherapy. However, there may be a subset of patients who are suitable for secondary debulking surgery at the time of recurrence.

The objectives of follow‐up include:

- Early recognition and prompt management of treatment‐related complications, including provision of psychological support.
- Early detection of symptoms or signs of recurrent disease.
- Collection of data regarding the efficacy of any treatment and the complications associated with those treatments in patients treated in clinical trials.
- Promotion of healthy behavior, including screening for breast cancer in patients with early‐stage disease, and screening for cervical cancer in patients having conservative surgery.

There are no evidence‐based guidelines regarding the appropriate follow‐up schedule. During the first year following treatment, patients are seen every 3 months with a gradual increase in intervals to every 4–6 months after 2 years and then annually after the fifth year. At each follow‐up, the patient should have her history retaken, including any change in family history of cancers and attention to any symptoms that could suggest recurrence; a physical and pelvic examination should be performed. This is an opportunity to refer appropriate patients for genetic testing if it was not done at diagnosis or during treatment. CA125 has traditionally been checked at regular intervals, but there has been debate regarding the clinical benefit of using CA125 progression alone as a trigger for initiating second‐line chemotherapy. A large MRC OV05‐EORTC 55955 study showed that treating asymptomatic patients with recurrent ovarian cancer with chemotherapy on the basis of CA125 progression alone did not improve survival and early treatment in asymptomatic patients had a negative impact on quality of life.^112^ This study has generated considerable debate regarding the use of CA125 for follow‐up, but most agree that it is reasonable not to immediately initiate treatment unless there is a clear clinical indication to do so. The timing of treatment should be based on symptoms as well as clinical and radiological findings. Imaging tests such as ultrasonography of the pelvis, CT, MRI, and/or positron emission tomography (PET) scans should be performed only when the clinical findings or the tumor markers suggest possible recurrence.

There appears to be no benefit to initiating chemotherapy in an asymptomatic patient with recurrent disease based only on rising CA125 levels in the absence of clinical symptoms or radiological evidence of recurrence. In asymptomatic patients with small volume disease and no radiological evidence of recurrence, close observation is a reasonable option, as well as entry into an appropriate clinical trial or possibly a trial of tamoxifen may be considered.

A Cochrane database systematic review of tamoxifen in unselected women with recurrent ovarian cancer reported a 10% objective response and a 32% disease stabilization rate.^113^ The patients treated were heterogeneous and included asymptomatic patients with rising CA125 levels, and symptomatic patients with chemotherapy‐resistant disease who had been heavily pretreated and had a poor performance status. GOG 198 compared tamoxifen and thalidomide in women with recurrent Stage III or IV epithelial ovarian, tubal, or peritoneal cancer who had completed first‐line chemotherapy, and who subsequently had Gynecologic Cancer InterGroup (GCIG) documented CA125 progression. The study reported that women who received thalidomide had a 31% increased risk of disease progression (HR 1.31), compared with those who were given tamoxifen.^114^ The median progression‐free survival was 3.2 months in the thalidomide group versus 4.5 months in the tamoxifen group. This suggests that tamoxifen may have a role in selected patients with a rising CA125 level, and the relationship between estrogen receptor positivity and benefit of tamoxifen in this patient population is being evaluated in current studies. In the PARAGON trial the role of anastrozole in 54 asymptomatic patients with rising CA125 was investigated in a phase 2 design.^115^ The primary endpoint was clinical benefit at 3 months and this was observed in 18 patients (34.6%; 95% CI, 23%–48%). The median duration of clinical benefit was 6.5 months (95% CI, 2.8–11.7). Most patients progressed within 6 months of starting anastrozole but 12 (22%) continued treatment for longer than 6 months. The role of hormonal therapy in this setting remains uncertain.

## 9 CHEMOTHERAPY FOR RECURRENT EPITHELIAL CANCER OF THE OVARY, FALLOPIAN TUBE, AND PERITONEUM

The majority of patients who present with advanced epithelial cancers of the ovary, fallopian tube, and peritoneum will relapse with a median time to recurrence of 16 months. Patients with recurrent ovarian cancer constitute a heterogeneous group with a variable prognosis, and a variable response to further treatment. The most widely used clinical surrogate for predicting response to subsequent chemotherapy and prognosis has been the progression‐free interval or the “platinum‐free interval,” which is defined as the time from cessation of primary platinum‐based chemotherapy to disease recurrence or progression.^116^, ^117^ This has been useful to define specific patient populations, but it has a number of limitations and depends on how patients are followed. In particular, it depends on how recurrence is detected and defined. Patients with a treatment‐free interval of less than 6 months are classified as platinum resistant and generally treated with nonplatinum‐based chemotherapy, while those with a treatment‐free interval of more than 6 months are considered to be platinum sensitive and commonly treated with platinum‐based chemotherapy. Patients who progress while on treatment or within 4 weeks of stopping chemotherapy are classified as platinum refractory.^116^, ^117^

There have been modifications to these definitions, and time to progression or recurrence rather than treatment‐free interval or platinum‐free interval has been used to define specific patient populations. There has been significant change in practice over the last 20 years and patients have been routinely followed with regular CA125 testing after completion of chemotherapy. For example, the “platinum‐resistant” subgroup may include asymptomatic patients with CA125 progression alone at 3 months post chemotherapy or radiological evidence of recurrence as well as those who are symptomatic with clinical recurrence. The Fourth Ovarian Cancer Consensus Conference reached agreement that distinct patient populations should be based on the interval from last platinum therapy and the time to progression. The progression‐free interval is defined from the last date of platinum dose until progressive disease is documented.^116^, ^117^

For patients whose disease is considered platinum sensitive, the ICON4 study showed advantage in terms of overall survival and progression‐free survival for a combination of carboplatin and paclitaxel versus single‐agent carboplatin.^118^ **Level of Evidence A**.

For patients with neurotoxicity, gemcitabine^119^ or liposomal doxorubicin^120^ may be substituted for paclitaxel. A large GCIG study (CALYPSO) compared carboplatin and liposomal doxorubicin (CD) with carboplatin and paclitaxel (CP) in 976 patients.^121^ The CD arm had statistically superior progression‐free survival compared with the CP arm, with a median progression‐free survival of 11.3 months versus 9.4 months, respectively. There was no significant difference in the overall survival between the treatment groups. Median overall survival was 33 months versus 30.7 months for the CP and CD arms, respectively. The CD arm was better tolerated with less severe toxicities, and this combination is now widely used. **Level of Evidence A**.

There is evidence that the addition of bevacizumab to the regimen of carboplatin and gemcitabine improves progression‐free survival compared with carboplatin and gemcitabine in platinum‐sensitive disease. In the OCEANS study,^122^ 484 patients with platinum‐sensitive disease were randomly assigned to carboplatin (AUC 4 on day 1) and gemcitabine 1000 mg/m^2^ on days 1 and 8) with or without bevacizumab (15 mg/kg on day 1) every 21 days cycles. Bevacizumab could be given concurrently with chemotherapy for a maximum of 10 cycles followed by bevacizumab alone until progression of disease or toxicity. The addition of bevacizumab to carboplatin and gemcitabine resulted in an improvement in progression‐free survival (12 months vs 8 months; HR 0.48; 95% CI, 0.39–0.61); however, there was no difference in overall survival between the two arms. Treatment with bevacizumab was associated with higher rates of serious hypertension (17% vs <1%), proteinuria grade 3 or higher (9% vs 1%), and noncentral nervous system bleeding (6% vs 1%).^122^ The OV21 trial randomized 682 patients with platinum‐sensitive recurrent ovarian cancer to 6 intravenous cycles of bevacizumab (15 mg/kg, day 1) plus carboplatin (AUC 4, day 1) plus gemcitabine (1000 mg/m^2^, days 1 and 8) every 3 weeks (standard group) or 6 cycles of bevacizumab (10 mg/kg, days 1 and 15) plus carboplatin (AUC 5, day 1) plus pegylated liposomal doxorubicin (30 mg/m^2^, day 1) every 4 weeks (experimental group), both followed by maintenance bevacizumab (15 mg/kg every 3 weeks in both groups) until disease progression or unacceptable toxicity. The median progression‐free survival was 13.3 months (95% CI, 11.7–14.2) in the experimental group versus 11.6 months (95% CI, 11.0–12.7) in the standard group (HR 0.81; 95% CI, 0.68–0.96; *P* = 0.012).^123^ The results of this trial support the experimental regimen in clinical practice.

For patients with definite platinum‐resistant disease, enrollment on available clinical trials or treatment with nonplatinum chemotherapy should be considered. There are a number of chemotherapy options including liposomal doxorubicin,^124^ topotecan,^124^ etoposide,^125^, ^126^ and gemcitabine.^127^, ^128^ The reported response rates are low, about 10%, with a median time to progression of 3–4 months and a median survival of 9–12 months. There have been many trials carried out with new agents in patients with platinum‐resistant ovarian cancer, including epothilones,^129^ trabectedin,^130^ and pemetrexed,^131^ among others, with no significant increase in response rates or progression‐free survival. More recently there have been encouraging reports of novel new agents or combinations including Wee1 (WEE1hu) inhibitor adavosertib combined with gemcitabine,^132^ as well as mirvetuximab soravtansine in patients with high folate receptor alpha expression,^133^ and these drugs are actively being investigated. There are many clinical trials in progress for patients with platinum‐resistant ovarian cancer and these are listed on ClinicalTrials.gov. No new cytotoxic agent has been approved to treat recurrent ovarian cancer for many years.

There is a role for angiogenesis inhibitors in platinum‐resistant ovarian cancer. In the AURELIA trial, women with recurrent platinum‐resistant ovarian cancer were randomized to standard of care, i.e. weekly topotecan, weekly paclitaxel, or monthly liposomal doxorubicin versus these agents combined with bevacizumab (10 mg/kg every 2 weeks, or 15 mg/kg every 3 weeks).^134^ Patients in the experimental arm had a longer progression‐free survival of 6.7 months versus 3.4 months and a higher overall response rate of 30.9% versus 12.6%. An exploratory subgroup analysis noted an increase in overall survival for weekly paclitaxel plus bevacizumab from 13.4 months to 22.4 months (with and without bevacizumab).^135^ The findings in the AURELIA trial changed the standard of care.

### 9.1 Immune checkpoint inhibitors in recurrent ovarian cancer

There has been much interest in exploring the role of immune checkpoint inhibitors in patients with recurrent ovarian cancer including those with platinum resistance. However, in general the results of these studies have been disappointing with low response rates reported. For example, KEYNOTE‐100 evaluated pembrolizumab, an anti‐PD‐1 antibody, in patients with recurrent ovarian cancer after multiple prior lines.^136^ The overall response rate was 8%, with a combined positive score (CPS, quantifying the number of PD‐L1 positive cells) over 10 the objective response rate was 11%–18%. Similarly, the response rate with avelumab, an anti‐PD‐L1 antibody, was 10% in recurrent ovarian cancer.^137^ However, there may be a role for combination regimens, which are being explored. For example, the phase 1/2 TOPACIO trial using niraparib and pembrolizumab in recurrent platinum‐resistant ovarian cancer showed a response rate of 18%.^138^ The combination of the CTLA‐4 antibody ipilimumab with nivolumab, an anti‐PD‐1 antibody induction, followed by nivolumab maintenance had an objective response rate of 31.4% compared with 12.2% with nivolumab alone in a recently reported randomized phase 2 trial.^139^ Although the median progression‐free survival was longer with combination, it was only 3.9 months versus 2 months, and the benefit questionable given the increased toxicity. The multicohort Leap‐005 trial recently reported preliminary data on another combination treatment using pembrolizumab and the multityrosine kinase inhibitor lenvatinib. In 31 patients with recurrent ovarian cancer the response rate was 29%.^140^ There are still more trials in progress that are likely to provide results over the next few years. It will take time to define the role of immune checkpoint inhibitors in patients with recurrent ovarian cancer, but it seems likely that only a small subset of patients benefits and the challenge is to identify who these patients are.

The optimal management of a patient with platinum‐resistant or refractory disease is complex and requires a careful assessment of the patient's performance status, symptoms, and extent of disease. Attention to symptom control and good palliative care is an essential component of management.

With very few exceptions, recurrent disease is not curable and the aim of treatment is to maintain quality of life and palliate symptoms particularly in patients with platinum‐resistant ovarian cancer.^141^ There are many potential treatment options, including chemotherapy, angiogenesis inhibitors, radiation therapy, or surgery in selected patients and inclusion in clinical trials. There is a subset of patients who may benefit from secondary surgical debulking.

### 9.2 PARP inhibitors as monotherapy in patients with recurrent ovarian cancer

Olaparib is US Food and Drug Administration (FDA) approved for the treatment of patients with germline BRCA‐mutated recurrent ovarian cancer who have received three or more prior lines of chemotherapy. The FDA granted approval on the basis of the response rate in a single‐arm study of olaparib in patients with BRCA mutations and with a wide range of different cancers. The response rate was 34% in heavily pretreated BRCA‐positive patients with platinum‐resistant recurrent ovarian cancer and the median progression‐free survival was 7.9 months.^142^

Rucaparib is also approved for treatment of BRCA‐mutation‐associated advanced ovarian cancer after completion of treatment with two or more chemotherapy regimens regardless of whether patients are platinum sensitive or resistant. Rucaparib's approval was based primarily on efficacy data from 206 patients with BRCA‐associated recurrent ovarian cancer who had prior treatment with two or more chemotherapy regimens and safety data from 377 patients with ovarian cancer treated with 600 mg rucaparib orally twice daily.^143^ Investigator‐assessed objective response rate was 54% and the median duration of response was 9.2 months.^143^

## 10 MANAGEMENT OF LOW‐GRADE SEROUS CANCERS

Low‐grade serous cancers (LGSCs) comprise 5%–10% of serous ovarian cancers and up to 8% of all ovarian cancers.^144^ They are typically diagnosed at a younger age than in women with high‐grade serous ovarian cancer (HGSOC), with a median age of 47–54 years at diagnosis, and are characterized by a relatively indolent behavior and resistance to cytotoxic chemotherapy.^145^ In contrast to HGSOC they do not have *TP53* mutations, but may have *KRAS* or *BRAF* mutations, and activation of the Ras‐Raf‐MEK‐ERK signaling pathway.^146^, ^147^

Most patients with low‐grade serous ovarian cancer (LGSOC) have advanced stage disease at initial diagnosis and the surgical management is similar to patients with high‐grade cancers, with attempts at total resection of tumor—with the exception of fertility‐sparing surgery in younger women with tumors confined to the ovary. Neoadjuvant platinum‐based chemotherapy for advanced stage LGSOC was associated with a radiological response rate of 4%, which is much lower than response rates of up to 80% in patients with HGSOC.^148^ Similarly, the response rates to chemotherapy have been reported to be low in a number of studies and the rate was only 3.7% (4.9% in patients with platinum‐sensitive disease and 2.1% in those with platinum‐resistant disease) in a report of patients with recurrent LGSC.^145^ A retrospective, exploratory, case–control analysis of over 5000 patients receiving adjuvant chemotherapy in clinical trials included 145 patients (2.8%) with LGSOC, of whom 37 had suboptimal debulking and were evaluable for response evaluation.^149^ The response rate was higher than other studies at 23.1% in this small subset of patients with LGSOC compared with 90.1% in patients with HGSOC.

Hormonal therapy has been reported to be associated with clinical benefit in recurrent and metastatic LGSC. Hormonal therapy was reported to have a response rate of 9% in a retrospective analysis of 64 patients with recurrent LGSC.^150^ In 26 patients with LGSC of the ovary or peritoneum, adjuvant hormone therapy following debulking surgery was associated with a median progression‐free survival of 22 months and a recurrence rate of 14.8%.^151^ In this small study, survival of the patients treated with adjuvant hormonal therapy was not significantly different to an age‐ and stage‐matched control group of patients with LGSC treated with surgery and adjuvant chemotherapy. A retrospective analysis was reported of 203 patients with LGSC of the ovary or peritoneum who received either maintenance/adjuvant hormonal treatment or observation, based on physician discretion, following primary cytoreductive surgery and platinum‐based chemotherapy.^152^ Patients who received adjuvant hormonal therapy had significantly longer median progression‐free survival (64.9 months vs 26.4 months) compared with the patients in the observation group, without significant prolongation of overall survival (115.7 months vs 102.7 months). The role of maintenance/adjuvant hormonal therapy in patients with LGSC will soon be tested in a large NRG Oncology trial.

LGSCs commonly show mutations in the MAP kinase pathway, particularly in *BRAF*, *KRAS* and *NRAS*. In view of this there have been a number of studies exploring targeted therapy with MEK inhibitors (MEKi). In a GOG phase 2 trial (GOG 0239) of the MEKi selumetinib in 52 women with recurrent LGSC, the overall response rate was 15%, with one complete response and seven partial responses with 65% of patients having stable disease.^153^ The median progression‐free survival was 11.0 months. The MILO trial was an open‐label phase 3 trial that randomized patients with recurrent LGSC to either chemotherapy (physician's choice of pegylated liposomal doxorubicin, paclitaxel, or topotecan) or MEK162 (binimetinib). This trial was stopped after a planned interim analysis showed that the hazard ratio for progression‐free survival crossed the predefined futility boundary.^154^ The median progression‐free survival was 9.1 months (95% CI, 7.3–11.3) for binimetinib and 10.6 months (95% CI, 9.2–14.5) for chemotherapy (HR1.21; 95% CI, 0.79–1.86), resulting in early study closure after 341 patients had enrolled. Secondary efficacy end points were similar in the two groups: overall response rate 16% versus 13% and median overall survival 25.3 months versus 20.8 months for binimetinib and chemotherapy, respectively. More recently a randomized trial (NRG‐GOG 0281) of the MEK inhibitor trametinib versus chemotherapy reported an improved objective response rate of 26.2% versus 6.2% in recurrent LGSC of trametinib compared with standard chemotherapy. In addition, the median progression‐free survival increased from 7.2 months with chemotherapy to 13 months with trametinib and overall survival was also increased, although this was not statistically significant.^155^ This remains an area of active investigation.

Follow‐up of patients with no evidence of disease is the same as for those with malignant epithelial carcinomas, but at less frequent intervals. **Level of Evidence C**.

### 10.1 Management of low malignant potential (borderline) tumors

Compared with invasive epithelial cancers, borderline tumors tend to affect a younger population and constitute 15% of all epithelial tumors of the ovary.^156^ Nearly 75% of these are Stage I at the time of diagnosis. The following can be said for these tumors^157^:

- The diagnosis must be based on the pathology of the primary tumor.
- Extensive sectioning of the tumor is necessary to rule out invasive cancer.
- The prognosis of these tumors is extremely good, with a 10‐year survival of about 95%.
- Invasive cancers that arise in borderline tumors are often indolent and generally have a low response to platinum‐based chemotherapy.
- Spontaneous regression of peritoneal implants has been observed.
- Early stage, serous histology, and younger age at diagnosis are associated with a more favorable prognosis.
- Although gross residual disease after primary laparotomy is associated with poorer prognosis, mortality from the disease remains low.
- Those patients who have invasive implants in the omentum or other distant sites are more likely to recur earlier. The role of cytotoxic chemotherapy is questionable as the response rates are low.

The causes of death include complications of disease (e.g. small bowel obstruction) or complications of therapy, and only rarely malignant transformation. The mainstay of treatment is primary surgical staging and cytoreduction. For patients with Stage I disease who want to preserve fertility, conservative surgery with unilateral salpingo‐oophorectomy can be considered after intraoperative inspection of the contralateral ovary to exclude involvement.^158^ For patients with only one ovary, or bilateral cystic ovaries, a partial oophorectomy or cystectomy can be considered for fertility preservation. For all other patients, total hysterectomy and bilateral salpingo‐oophorectomy are recommended, with maximal cytoreduction if the disease is metastatic.

Patients with borderline tumors in all stages of disease should be treated with surgery. A small percentage of patients with invasive implants may respond to chemotherapy but the response to chemotherapy is low. Uncommonly, some patients recur early and have higher‐grade invasive cancers and may benefit from chemotherapy.^159^

In patients with late recurrence of the disease, secondary cytoreduction should be considered, and chemotherapy given only if invasive disease is present histologically.

Follow‐up of patients with no evidence of disease is the same as for those with malignant epithelial carcinomas, but at less frequent intervals. If the contralateral ovary has been retained, it should be followed by transvaginal ultrasonography, at least on an annual basis.^1^, ^157^, ^160^ **Level of Evidence C**.

## 11 MANAGEMENT OF GRANULOSA CELL TUMORS

Granulosa cell tumors account for about 70% of sex‐cord stromal tumors and 3%–5% of all ovarian neoplasms.^2^ There are two types of granulosa cell tumors: the juvenile and the adult types. Because of the high estrogen production, the juvenile type typically presents with sexual precocity, while the adult type may present with postmenopausal bleeding. The majority of patients are diagnosed with Stage I tumors. The peak incidence is in the first postmenopausal decade.^2^, ^161^

Granulosa cell tumors are generally indolent (i.e. with a tendency to late recurrence). Stage at diagnosis is the most important prognostic factor. Other prognostic factors include age at diagnosis, tumor size, and histologic features. If metastatic, adequate cytoreduction is the mainstay of treatment. If the patient is young and the disease is confined to one ovary, conservative surgery should be performed.^162^, ^163^

Infrequency of the disease and its protracted course has resulted in a lack of prospective studies. There is no evidence that adjuvant chemotherapy or radiotherapy improves the results of surgery alone for Stage I disease. The value of postoperative adjuvant chemotherapy for higher‐risk Stage I disease (tumor size >10 cm, capsule rupture, high mitotic count) is uncertain, and has not been tested in randomized studies. Platinum‐based chemotherapy is used for patients with advanced or recurrent disease, with an overall response rate of 63%–80%.^163^, ^164^, ^165^ Bleomycin/etoposide/cisplatin (BEP) has been widely used to treat patients with metastatic granulosa cell tumors; however, there is significantly increased toxicity of bleomycin in patients over the age of 40 years and there were a number of deaths associated with bleomycin in early GOG trials, which led them to reduce the bleomycin dose to 20 units/m^2^ intravenously every 3 weeks (x 4) to reduce toxicity.^166^ Carboplatin and paclitaxel appear to have a similar response rate and less toxicity than BEP.^165^ The optimal chemotherapy regimen is an open question and is being addressed in GOG‐0264 (NCT01042522), which is randomizing patients with recurrent/metastatic granulosa cell tumors to BEP or carboplatin and paclitaxel.

Bevacizumab has also been reported to have single‐agent activity with a response rate of 16% in 36 patients with granulosa cell tumors and measurable disease.^167^ ALIENOR/ENGOT‐ov7 is a randomized phase 2 trial that compared weekly paclitaxel with weekly paclitaxel in combination with bevacizumab in 60 patients with relapsed granulosa cell tumors. The overall response rate increased with the addition of bevacizumab (25% with weekly paclitaxel vs 44% with the combination), but there was no statistical difference in the primary endpoint; progression‐free survival at 6 months was 71% (55–84%) and 72% (55–87%) in the weekly paclitaxel and weekly paclitaxel with bevacizumab arms, respectively.^168^

Hormonal therapies have also been widely used to treat patients with recurrent granulosa cell tumors. A systematic review of hormonal therapies that included retrospective studies with a total of 31 patients reported overall response rates of 71%.^169^ A retrospective single‐center series of 15 patients treated with letrozole reported a partial response rate of 41% and a median progression‐free survival of over 20 months. In contrast, a retrospective study that included 22 patients with evaluable disease reported a response of 18% and 64% had stable disease with an aromatase inhibitor.^170^ PARAGON is the only prospective trial and reported a 10.5% partial response rate with anastrozole but a high proportion of patients with stable disease.^171^ It is not clear if the stable disease is due to treatment or the indolent biology of granulosa cell tumors.

Follow‐up is clinical. For patients with elevated levels of inhibin B and/or anti‐müllerian hormone at initial diagnosis of granulosa cell tumors, inhibin B and/or anti‐müllerian hormone appear to be reliable markers during follow‐up for early detection of residual or recurrent disease.^172^

There is no evidence‐based preference for inhibin B or anti‐müllerian hormone as a tumor marker.^173^ Serum inhibin is a useful tumor marker in postmenopausal women. **Level of Evidence C**.

## 12 MANAGEMENT OF GERM CELL MALIGNANCIES

This group of ovarian tumors consists of a variety of histologically different subtypes that are all derived from the primitive germ cells of the embryonic gonad. Malignant germ cell tumors represent a relatively small proportion of all ovarian tumors. Prior to advances in chemotherapy, the prognosis for these aggressive tumors was poor. The use of platinum‐based chemotherapeutic regimes has made germ cell malignancies among the most highly curable cancers.^161^

### 12.1 Presentation

These are most common ovarian tumors in the second and third decades of life. They are frequently diagnosed by finding a palpable abdominal mass in a young woman who complains of abdominal pain. The following are the symptoms of germ cell tumors in order of frequency^161^:

- Acute abdominal pain.
- Chronic abdominal pain.
- Asymptomatic abdominal mass.
- Abnormal vaginal bleeding.
- Abdominal distention.

### 12.2 Histologic classification

The classification of germ cell tumors of the ovary is important to determine prognosis and for treatment with chemotherapy. Germ cell tumors are classified as follows^2^, ^161^:

- Dysgerminoma.
- Embryonal carcinoma
- Teratoma (immature, mature, mature with carcinoma [squamous cell, carcinoid, neuroectodermal, malignant struma, etc]).
- Extra‐embryonal differentiation (choriocarcinoma, endodermal sinus tumor [yolk sac tumor]).

### 12.3 Diagnosis, staging, and surgical management

Ovarian germ cell tumors are staged similarly to epithelial carcinomas, although the staging system used for male germ cell tumors is probably more useful. The approach to treatment is based on the principles of management of metastatic germ cell tumors of the testis (i.e. low, intermediate, and poor risk). Dysgerminoma is the equivalent of seminoma in testicular cancer.^174^ It is exquisitely sensitive to platinum‐based chemotherapy and is radiosensitive. The cure rate is high irrespective of the stage. The other histologic subtypes are equivalent to nonseminomatous testicular cancer. The aggressiveness of the disease is dependent on the type—the most aggressive being endodermal sinus and choriocarcinoma, but with combination chemotherapy, they are highly curable.^175^, ^176^, ^177^, ^178^, ^179^

As chemotherapy can cure the majority of patients, even with advanced disease, conservative surgery is standard in all stages of all germ cell tumors. Conservative surgery means laparotomy with careful examination and biopsy of all suspicious areas, with limited cytoreduction, thereby avoiding major morbidity. The uterus and the contralateral ovary should be left intact. Wedge biopsy of a normal ovary is not recommended as it defeats the purpose of conservative therapy by potentially causing infertility. Patients with advanced disease may benefit from 3–4 cycles of neoadjuvant chemotherapy using BEP (bleomycin, etoposide, cisplatin [platinum]) regimen with preservation of fertility.^180^ Patients who receive conservative surgery with the preservation of one ovary retain acceptable fertility rates despite adjuvant treatment with chemotherapy. There has been no report of higher adverse obstetric outcome or long‐term unfavorable sequelae in the offspring.

Secondary surgery is of no proven benefit, except in those patients whose tumor was not completely resected at the initial operation and who had teratomatous elements in their primary tumor. Surgical resection of residual masses may be beneficial in such patients, as there may be mature teratomatous nodules that can continue to increase in size (growing teratoma syndrome), and more rarely can undergo malignant transformation over time to an incurable malignancy (e.g. squamous cell carcinoma).^181^

### 12.4 Postoperative management and follow‐up of dysgerminoma

Patients with Stage IA disease may be observed after surgery. A small proportion of patients may recur, but they can be treated successfully at the time of recurrence with a high rate of cure. Patients with disease beyond the ovary should receive adjuvant chemotherapy. Although radiation therapy is effective, it is no longer used in view of late effects and chemotherapy is highly effective.

A follow‐up surveillance regime for patients with Stage IA dysgerminoma is outlined in Table 5. This schedule is based on the experience managing seminomas in males and the reports by Dark et al.^182^ and Patterson et al.^183^ This pragmatic follow‐up schedule and has not been tested in randomized trials.

**TABLE 5:** Follow‐up regime for Stage I germ cell malignancies^a^

Regimen	Description
Surveillance	Baseline CT chest, abdomen, and pelvis, if not performed preoperatively
	Repeat CT or MRI, abdomen and pelvis at 3 months after surgery
	Repeat CT or MRI abdomen plus pelvis at 12 months
	Pelvic ultrasound alternate visits (not when having CT scan) for 2 years if nondysgerminoma and for 3 years if dysgerminoma
	Chest X‐ray at alternate visits
Clinical examination	
1 year	Monthly
2nd year	2 monthly
3rd year	3 monthly
4th year	4 monthly
Years 5–10	6 monthly
Tumor marker follow‐up	Samples: serum AFP and hCG, LDH and CA 125 (regardless of initial value)
0–6 months	2 weekly
7–12 months	4 weekly
12–24 months	8 weekly
24–36 months	12 weekly
36–48 months	16 weekly
48+ months	6 monthly until year 10

Abbreviations: AFP, alpha‐fetoprotein; hCG, human chorionic gonadotropin; LDH, lactate dehydrogenase.

^a^ Adapted from Patterson et al.^183^

#### 12.4.1 Chemotherapy for dysgerminoma

Dysgerminoma is extremely sensitive to chemotherapy and treatment with chemotherapy cures the majority of patients, even with advanced disease.^161^, ^184^ The recommended chemotherapy regimen is as follows:

- Etoposide (E) 100 mg/m^2^ IV per day for 5 days every 3 weeks for 3 cycles.
- Cisplatin (P) 20 mg/m^2^ IV per day for 5 days every 3 weeks for 3 cycles.
- Bleomycin (B) 30 IU IV/IM on days 1/8/15 for 12 weeks (optional) (Note: bleomycin is dosed in International Units). If bleomycin is omitted, then 4 cycles of EP are commonly used. (Note that various schedules of bleomycin have been used and the role of bleomycin in dysgerminomas is controversial).

There is increased interest in de‐escalation of chemotherapy in dysgerminomas as they are so chemosensitive. It may be possible to omit bleomycin and substitute carboplatin for cisplatin due to the acute adverse effects and potential long‐term adverse effects associated with BEP, which include secondary malignancies, cardiovascular disease, hypertension, Raynaud's phenomenon, pulmonary toxicity, nephrotoxicity, neurotoxicity, deafness, decreased fertility, and psychosocial problems amongst others. GOG 116 is an old trial that investigated carboplatin 400 mg/m^2^ and etoposide 120 mg/m^2^ on days 1–3 every 4 weeks in 39 patients with Stage IB–III dysgerminoma.^185^ No patients relapsed despite the very modest dose of carboplatin and 3 days of etoposide every 4 weeks for 3 cycles only; but the trial closed early after the results of two trials in males with nonseminomatous testicular cancer reported inferior outcomes with carboplatin compared with cisplatin. Shah et al.^186^ reported the results of pooled data from six trials (three pediatric and three adult) on behalf of the Malignant Germ Cell Tumor International Consortium (MaGIC), which included 126 patients with advanced stage (Stage IC–IV) dysgerminomas who were treated with either carboplatin‐ or cisplatin‐based chemotherapy. Survival outcomes were equivalent with a 96% 5‐year survival in both groups with no differences seen according to age (<25 or >25 years old). Seven patients relapsed including two patients treated with carboplatin‐based chemotherapy and five with BEP, and all were salvaged.

When there is bulky residual disease it is common to give 3–4 courses of BEP or EP chemotherapy.^187^ **Level of Evidence B**.

The optimal follow‐up schedule has not been clinically investigated in ovarian germ cancers and the frequency of visits and investigations is controversial. Patients who have Stage I tumors and are offered surveillance need to be seen regularly and one option is to utilize the follow‐up regimen presented above.^182^ Patients who have had chemotherapy have a lower risk of recurrence and the frequency of CT scans can be reduced, which is similar to the approach for testicular germ cell tumors.^183^ Each follow‐up visit should involve taking a medical history, physical examination, and tumor marker determination. Although tumor markers are important, radiological imaging is also pertinent, especially for patients whose tumor markers were not raised at diagnosis. CT or MRI scans should be performed as clinically indicated.^182^

Patients who have not received chemotherapy should be followed closely. Ninety percent of relapses in these patients occur within the first 2 years. At relapse, with few exceptions, these patients can be successfully treated.^182^ **Level of Evidence D**.

### 12.5 Postoperative management and follow‐up of nondysgerminoma germ cell malignancies

These tumors are highly curable with chemotherapy, even with advanced disease. Patients with Stage IA grade 1–2 immature teratoma have a very good prognosis and should be only observed after primary conservative surgery. Adjuvant chemotherapy does not appear to add any survival benefit in this subgroup of patients. Although adjuvant chemotherapy has been routinely recommended to all other patients with Stage I nondysgerminomatous ovarian germ cell tumors, this approach has been challenged and there may be a role for close surveillance and chemotherapy reserved for the subset who relapse as this is the standard of care in males with apparent Stage 1 testicular cancers. All other patients with nondysgerminomas, and higher‐stage and higher‐grade immature teratomas, should receive postoperative adjuvant chemotherapy.^161^

The recommended chemotherapy regimen is etoposide 100 mg/m^2^ per day for 5 days with cisplatin 20 mg/m^2^ per day for 5 days, and bleomycin at 30 IU IM/IV on days 1, 8, and 15 for a total of 12 weeks of treatment. For patients with good prognosis disease, 3 cycles of BEP are recommended, while patients with intermediate/poor risk disease should receive 4 cycles of BEP.^161^

Patients who relapse after BEP may still attain a durable remission and cure with second‐line chemotherapy regimens such as paclitaxel–ifosfamide–cisplatin (TIP).^177^ High‐dose chemotherapy and autologous marrow rescue may be considered in selected patients. These patients should be managed in specialized units.

After chemotherapy, patients with metastatic immature teratomas can sometimes have residual masses, which are composed entirely of mature elements. These masses can grow (growing teratoma syndrome), and should be resected after the completion of chemotherapy.^188^ **Level of Evidence B**.

All patients should have alpha‐fetoprotein (AFP) and human gonadotropin (beta hCG) to monitor response to treatment. All patients treated with chemotherapy should be followed up with medical history, physical examination, and appropriate tumor markers in the same way as dysgerminomas. CT or MRI scans should be performed as clinically indicated.^159^

Relapses in patients usually occur within the first 2 years after diagnosis^161^, ^177^ **Level of Evidence D**.

## 13 SARCOMA OF THE OVARY

Ovarian sarcomas are rare and occur primarily in postmenopausal patients.^161^, ^189^ Nevertheless, accurate diagnosis and differentiation from other types of primary ovarian cancer are important, as the prognosis is generally poor.

There are two types of sarcoma. Malignant mixed Müllerian tumors (MMMTs) or ovarian carcinosarcomas, the more common of the two, are biphasic tumors composed of both carcinomatous and sarcomatous elements.^189^, ^190^ Most authors agree that most MMMTs are monoclonal in origin and should be thought of and managed as a high‐grade epithelial cancer. The sarcomatous component is derived from the carcinoma or from a stem cell that undergoes divergent differentiation. Thus, ovarian carcinosarcomas are best regarded as metaplastic carcinomas.

Pure sarcomas are very rare and should be treated according to the specific histologic subtype. These rare sarcomas include fibrosarcomas, leiomyosarcomas, neurofibrosarcomas, rhabdomyosarcomas, chondrosarcomas, angiosarcomas, and liposarcomas. Their management is not discussed here.

Patients with early stage MMMTs/ovarian carcinosarcomas have a better outcome than those with advanced stage disease, but the overall prognosis is poor. They should be managed similarly to high‐grade pelvic serous cancers. Their rarity prohibits any prospective randomized trials.

The principles of surgical management of ovarian MMMTS/ovarian carcinosarcomas are the same as for high‐grade pelvic serous cancers. Following surgery, patients should receive platinum‐based chemotherapy.^161^ The follow‐up schedule is as recommended for epithelial malignancies. **Level of Evidence C**.

## AUTHOR CONTRIBUTIONS

JB, MR, SK, LK, and MF reviewed and updated the chapter on cancer of the ovary, fallopian tube, and peritoneum published in the 2018 Cancer Report.

## CONFLICTS OF INTEREST

Outside the submitted work, JSB reports institutional research funding received from Tesaro and ImmunoGen, and participation on a Merck Data Safety Monitoring Committee (MK‐7339‐001 ENGOT‐ov43). Outside the submitted work, MF reports institutional research grants received from AstraZeneca, Novartis, and Beigene; consulting fees from AstraZeneca, GSK, MSD, Lilly, Novartis, and Takeda; honoraria for lectures from AstraZeneca, GSK, and ACT Genomics; and participation on an AGITG Data Safety Monitoring Board. All other authors report no conflicts of interest.
